# Lifestyle Interventions for Persons with Dementia: Singing your Way Toward Wellness

**DOI:** 10.1093/geroni/igaa057.3248

**Published:** 2020-12-16

**Authors:** Cynthia McDowell, Sebastian Santana, André Smith, Debra Sheets, Stuart MacDonald

**Affiliations:** University of Victoria, Victoria, British Columbia, Canada

## Abstract

Arts-based interventions represent an inexpensive, non-pharmacological, and non-invasive approach to help mitigate negative symptoms and improve quality of life for persons with dementia (PwD). The present study examined whether a social singing intervention can modulate patterns of cognitive change and whether select biopsychosocial indicators exhibit concomitant within-person time-varying covariation. Participants with dementia (n=32, mean age=79.6 years; 53% female) engaged weekly in the Voices in Motion project, an intergenerational, social-cognitive choral intervention spanning up to 18 months and 9 individual assessments. The Mini Mental State Examination (MMSE), gait velocity, and positive and negative affect were assessed using an intensive repeated-measures design, with multilevel models of change employed to disaggregate both between- and within-person effects. Across months of the social intervention, several significant within-person time-varying associations were observed; on occasions when a given individual performed one unit faster on gait velocity (p<.05) or one unit lower on negative affect (p<.01), relative to their personal average, there were corresponding improvements in cognitive function. Notably, in contrast, MMSE change remained relatively stable over the course of the 18-month intervention (-0.105, p=0.12), with little between-subject variability in rates of change. These findings imply that, within-persons, reducing comorbidities associated with dementia (e.g., elevated negative affect and its corresponding impact on cognitive resource competition) through participation in a lifestyle intervention may facilitate increases in cognitive, physiological, and psychological function. Implications are discussed with regard to the merits of invoking virtual lifestyle interventions for socially isolated individuals (e.g., PwD and those in residential care).

